# Estimating Social Variation in the Health Effects of Changes in Health Care Expenditure

**DOI:** 10.1177/0272989X20904360

**Published:** 2020-02-15

**Authors:** James Love-Koh, Richard Cookson, Karl Claxton, Susan Griffin

**Affiliations:** Centre for Health Economics, University of York, York, North Yorkshire, UK; Centre for Health Economics, University of York, York, North Yorkshire, UK; Centre for Health Economics, University of York, York, North Yorkshire, UK; Department of Economics and Related Studies, University of York, York, North Yorkshire, UK; Centre for Health Economics, University of York, York, North Yorkshire, UK

**Keywords:** distributional cost-effectiveness analysis, health equity, health inequality, health opportunity cost

## Abstract

**Background.** A common aim of health expenditure is to reduce unfair inequalities in health. Although previous research has attempted to estimate the total health effects of changes in health expenditure, little is known about how changes affect different groups in the population. **Methods.** We propose a general framework for disaggregating the total health effects of changes in health expenditure by social groups. This can be performed indirectly when the estimate of the total health effect has first been disaggregated by a secondary factor (e.g., disease area) that can be linked to social characteristics. This is illustrated with an application to the English National Health Service. Evidence on the health effects of expenditure across 23 disease areas is combined with data on the distribution of disease-specific hospital utilization by age, sex, and area-level deprivation. **Results.** We find that the health effects from NHS expenditure changes are produced largely through disease areas in which individuals from more deprived areas account for a large share of health care utilization, namely, respiratory and neurologic disease and mental health. We estimate that 26% of the total health effect from a change in expenditure would accrue to the fifth of the population living in the most deprived areas, compared with 14% to the fifth living in the least deprived areas. **Conclusions.** Our approach can be useful for evaluating the health inequality impacts of changing health budgets or funding alternative health programs. However, it requires robust estimates of how health expenditure affects health outcomes. Our example analysis also relied on strong assumptions about the relationship between health care utilization and health effects across population groups.

Two central objectives of publicly funding health care systems are to improve population health and to reduce health inequality. In England, for example, there is a legal obligation to consider reducing population health inequalities in determining which services to include within the National Health Service (NHS).^1^ Although many studies have examined the impact of health care expenditure on population health,^2–7^ less is known about the impact on health inequality.

Conventional benefit incidence studies have examined the average overall health care expenditure by sociodemographic group.^8^ However, if policy makers wish to reduce health inequality, evidence on the distribution of the benefits of additional investment in health care (i.e., the changes in health outcome in each group as expenditure is altered) is required. These marginal benefits can be compared with investment in other programs, such as education and social protection, or used to compare interventions within the health sector in terms of their impact on health inequality.

We propose a method for estimating the relationship between a change in overall health expenditure and changes in the social distribution of healthy life expectancy. This approach can estimate effects for a variety of sociodemographic characteristics simultaneously and can enable an analysis of the effects of expenditure on multiple dimensions of health inequality. We demonstrate this method with an application to England, using the results of a study of the marginal productivity of the English NHS.^9^

A further application is to use the estimated outputs in equity-informative cost-effectiveness analysis.^10^ The health benefits accruing to different social groups from a new intervention can be compared against the social distribution of health benefits that would be expected as a result of general health care expenditure to determine whether the intervention would reduce health inequalities by more than existing services. The results from our method describe the social distribution of health opportunity costs of health care expenditure that can be used to inform health care decision making.

## Methods

### Overview

We describe a general framework for estimating a social distribution of health effects from marginal changes in health care expenditure. This framework uses the results of marginal productivity studies that draw causal inferences about the relationship between health expenditure and health outcomes. Where the marginal productivity study does not directly estimate differences in marginal productivity between population groups, our framework shows how the estimated change in health from a given change in expenditure can be disaggregated indirectly. We illustrate the framework through an application to the English NHS, using evidence from a marginal productivity study for England.^9,11^ The social groups in our example are based on age, sex, and socioeconomic characteristics, but the method can in principle be applied to other variables considered relevant to assessing unfair differences in health. We hereafter refer to the characteristics that delineate the population groups as “equity-relevant” variables.

### Analytical Framework

The results of marginal productivity studies of health care expenditure describe the total health effect ($h^{T}$) from a given change in expenditure. Our framework addresses the question of how the health effects from the change in expenditure are distributed between equity-relevant social groups. We express this formally as the share of the total health effect that is received by each group (denoted $p_{x}$). Algebraically, the relationship between $h^{T}$ and $p_{x}$ can be written as $h^{T}=\sum_{x}h^{T}p_{x}=\sum_{x}h_{x},$ where $h_{x}$ is the outcome of interest, that is, the health change for each equity-relevant group x.

The distribution of marginal health effects ($h_{1},h_{2},\ldots ,h_{x}$) could be estimated directly within a marginal productivity analysis. Including an interaction term between health sector expenditure and an equity variable (i.e., area-level socioeconomic deprivation) in the statistical model that links expenditure to outcomes, or estimating separate models for each equity-relevant group, would yield separate health effects for each group. This would require data on equity-related characteristics at the level of the unit of analysis (e.g., by disease area and geography). No existing marginal productivity studies have so far estimated effects in terms of equity-relevant groups. This could be due to the lack of information on the equity-relevant characteristics in the data sets used in marginal productivity analysis or the additional challenges posed by including interaction terms in statistical models estimating causal effects.^12^

Indirect estimation of the distribution of health effects is instead possible if the marginal productivity study reports health effects in terms of a secondary factor (j) that can be linked to equity-relevant characteristics, such as health care facility type or disease area. The total marginal health effect can be defined as the sum of J subsidiary effects ($h_{j}$), such that $h^{T}=\sum_{J}h_{j}$. Each $h_{j}$ can then be split between equity-relevant groups (denoted $h_{xj}$), which are then summed over all the secondary groups to obtain the total health effect for each equity-relevant group:

$$h_{x}=\sum_{J}h_{xj}=\sum_{J}h_{j}p_{xj}$$

where $p_{xj}$ is the proportion of the health effect in category j accruing to equity-relevant group x. The proportion of the overall health effect accruing to each group can then be obtained from the group-specific health effects and the total health effect via the formula $p_{x}=\frac{h_{x}}{h^{T}}$.

The proportions $p_{x}$ provide the means to calculate the distribution of health effects for any given marginal change in health care expenditure (Δc). The change in expenditure is first converted into the total health effect ($h^{T|\Delta c}$) using the marginal cost of producing 1 additional unit of health (k). This summary measure of marginal productivity represents the rate at which health resources are converted into health at the margin. The group-specific effects ($h_{x|\Delta c}$) are obtained by multiplying the total health effect by the respective proportion $p_{x}$:

$$h_{x|\Delta c}=\Delta c/kp_{x}=h^{T|\Delta c}p_{x}$$

A worked example demonstrating these calculations is provided in online Supplementary Appendix A.

### Data and Assumptions

Applying the framework described above requires a reliable and valid estimate of health system marginal productivity. A number of these studies have been conducted since 2015,^9,13–15^ all of which employed an empirical strategy to exploit variation in health outcomes and health expenditure. This variation can be between geographical areas, over time, or a combination of the two. Identifying the causal effect of health sector expenditure on health outcomes is, however, empirically challenging.^16^ Reverse causality between outcomes and expenditure may be present, as historically poor health outcomes may lead to the allocation of extra health resources. An array of potentially unobservable environmental factors, with complex causal pathways, also determine health. For these reasons, studies investigating health system marginal productivity have adopted statistical methods that use instrumental variables to control for these unobserved factors.

A further challenge is that mortality data are most readily available as the basis for establishing a causal effect, but the impacts of health expenditure are not restricted to risk of death and for some diseases (e.g., hearing or vision) may be almost entirely in terms of health-related quality of life (HRQL). Marginal productivity studies have therefore developed methods for adjusting their mortality-based results to reflect HRQL effects.

To apply our framework, data linking the secondary factor to equity-relevant characteristics are needed. It is unlikely that data sources will able to validly allocate the health effects to equity-relevant groups directly, requiring assumptions to be made when they are used. For example, if the secondary factor is disease area, there is often evidence linking disease incidence or prevalence to equity-relevant characteristics. However, neither reflect differences in health care–seeking behavior between social groups. Allocating the health effects in each disease area using these data may therefore overestimate the share of health for any groups who are less likely to use services. This can be seen in the treatment of hepatitis C patients in the United Kingdom, for example, for whom uptake of services is lower in minority ethnic groups and intravenous drug users.^17^

Information describing differences in health care utilization in each disease area between equity-relevant groups does account for differences in uptake. However, simply using the distribution of utilization to allocate the health effect assumes that each particular episode of care generates the same health benefit, regardless of the social characteristics of the recipient. For example, in the disease area of cancer using the socioeconomic distribution of surgical removal of tumors to describe the distribution of the health benefits from surgery would assume that every individual achieves equal benefit from undergoing surgery regardless of socioeconomic status. These assumptions can sometimes be relaxed, for example, if there is evidence on the variation of health benefits from utilization between groups. If a group with high socioeconomic status was found to yield greater benefits relative to lower groups, this can be used to weight its respective share of the health benefits.

The information linking the secondary factor with equity-relevant characteristics should describe how different social groups are affected by changes in health expenditure at the margin. Using the previous example, we would ideally want to know the distribution of additional (i.e., marginal) utilization in each disease area following an increase in health expenditure. Trying to estimate this relationship between health expenditure and utilization shares many of the complexities encountered during the analysis of health system marginal productivity, such as reverse causality. Using the distribution of average utilization in the absence of information on marginal utilization will assume that the former adequately represents the latter. Empirical evidence on this topic is limited but indicates that the 2 distributions can differ.^18^

### Analyzing Inequalities

Inequality in marginal health effects can be explored with respect to each equity characteristic included in the analysis. There are numerous ways to summarize the extent of inequality in a distribution, and in general, we desire those that encompass both the magnitude and direction of inequality (i.e., whether it favors the “worst off” or the “best off” and by how much). Where the equity-relevant characteristic divides the population into 2 groups, absolute and relative differences in health outcomes can be calculated directly. The same can be done for categorical variables if it is appropriate to consider specific pairwise comparisons. To compare across large numbers of groups or a continuous measure that can be ordered from worst off to best off, a range of measures can be employed to summarize the differences (see Regidor^19^ for an overview). These include regression-based measures such as the slope index of inequality (SII) and the relative index of inequality (RII), respectively.

The SII is the slope coefficient from a regression analysis, in which the health effect ($h_{x}$) or the proportion of the health effect ($p_{x}$) of an expenditure change is the dependent variable and the equity characteristic of interest is the independent variable. The slope coefficient then describes the absolute difference in the share of health effects for a 1-unit increase in the equity variable. To make this easy to interpret, it can be helpful to adjust the equity variable to achieve a 0–1 scaling. This allows the SII to be interpreted as the difference between best off and worst off group. If, for example, the SII is estimated using income rank as the equity-relevant variable, then an SII of −0.2 would mean that the proportion of the overall health effect accruing to individuals at the bottom of the income distribution is 0.2 higher than for those at the top of the distribution. The RII is obtained by dividing the SII by the mean of the dependent variable. In the example using income rank, an RII of −1 implies that individuals in the lowest income group accrue double the health from a marginal change in expenditure as those in the top income group.

It is also possible to calculate health effects in terms of a third variable not included in the marginal productivity or indirect analysis. For example, a geographical distribution can be imputed using the sociodemographic characteristics of each region. If individuals with low socioeconomic status gain a higher share of health effects, for example, then the regions in which more socioeconomically deprived individuals live should also exhibit a higher share. This can be expressed as an index that is greater than 1 when the proportion of the health effect accruing to an area is greater than its respective share of the overall population, and vice versa. This is described further in Supplementary Appendix B.

### Case Study: Health Effects of NHS Spending in England

Our example uses the results of a study of the marginal productivity of the English NHS.^9^ The authors used cross-sectional data on health care expenditure and mortality across 152 regional spending bodies (primary care trusts) in England in 2008, broken down by 23 broad disease areas (such as cancer or respiratory illness) called “program budgeting categories” (PBCs). We used disease area as the secondary factor by which to link health effects to equity-relevant characteristics. An instrumental variables approach was used to account for endogeneity bias and has been validated by similar results in subsequent studies.^20,21^

The econometric models estimated the elasticity of mortality with respect to health care expenditure: the percentage change in mortality given a 1% change in expenditure. Mortality was measured in terms of years of life lost (YLL), the total number of life years lost in 1 year due to premature death for all diseases within a PBC. By combining the change in YLL across all disease areas from a given change in expenditure, the authors derived a marginal cost of £17663 per life year. To account for gains to quality of life as well as survival, further adjustments were made to obtain effects in terms of quality-adjusted life-years (QALYs). The QALY is a measure of health that accounts for both health-related quality of life and survival by weighting the time spent alive according the individual’s health state.^22^

Health effects in terms of QALYs were estimated by applying the elasticities from the YLL equations to the QALY burden associated with each PBC (the annual total of QALYs lost due to premature death and disability associated with all the diseases within a PBC). For example, a 1% change in NHS expenditure was estimated to yield a 1.6% change in YLL due to respiratory disease (PBC 11); applying this to the QALY burden of respiratory disease gives a change in QALYs of 17981. This represents 29.7% of the overall change of 60600 QALYs across all PBCs. The distribution of these QALYs over PBCs, which correspond to the quantities $h_{j}$ in our framework, are given in Table 1. An overview of the methods used by Claxton and colleagues can be found in Supplementary Appendix C.^9^

**Table 1:** Change in QALYs Generated from a 1% Change in NHS Expenditure in England by Disease Area

PBC No.	Disease Area	Health Effect (QALYs)	Proportion of Total Health Effect
	Total	60660	1
11	Respiratory	17981	0.2964
7	Neurological	8551	0.1410
10	Circulatory	8453	0.1394
5	Mental health	7469	0.1231
4	Endocrine	4749	0.0783
13	Gastrointestinal	3441	0.0567
2	Cancers and tumors	2064	0.0340
15	Musculoskeletal	1819	0.0300
3	Blood disorders	1712	0.0282
1	Infectious diseases	1229	0.0203
9	Hearing	1098	0.0181
17	Genito-urinary	829	0.0137
12	Dental	533	0.0088
8	Vision	333	0.0055
14	Skin	152	0.0025
20	Poisoning	64	0.0011
6	Learning disability	54	0.0009
21	Healthy individuals	53	0.0009
18 + 19	Maternity and neonate	18	0.0003

NHS, National Health Service; PBC, program budgeting category; QALY, quality-adjusted life-year. Source: Claxton et al.^9^

### Health Effects by Social Group

The social characteristics used in this example are age, sex, and socioeconomic status. The latter is defined by the Index of Multiple Deprivation (IMD), an area-based measure of socioeconomic deprivation. We estimated the proportion of the health effect accruing to each subgroup in 2 steps, starting with age and sex first and then socioeconomic status separately. The age and sex proportions came from a subsequent publication by Claxton and colleagues,^11^ in which the PBC-level effects were split by age and sex using disease incidence statistics for the United Kingdom, obtained from the World Health Organization’s Global Burden of Disease Study.^23^ For example, it was calculated that 6.1% of the incident population in the respiratory disease PBC are 30- to 44-year-old women, meaning that the health effect accruing to this group was estimated to be 17981 × 0.061 = 1097 QALYs.

**Figure 1:**
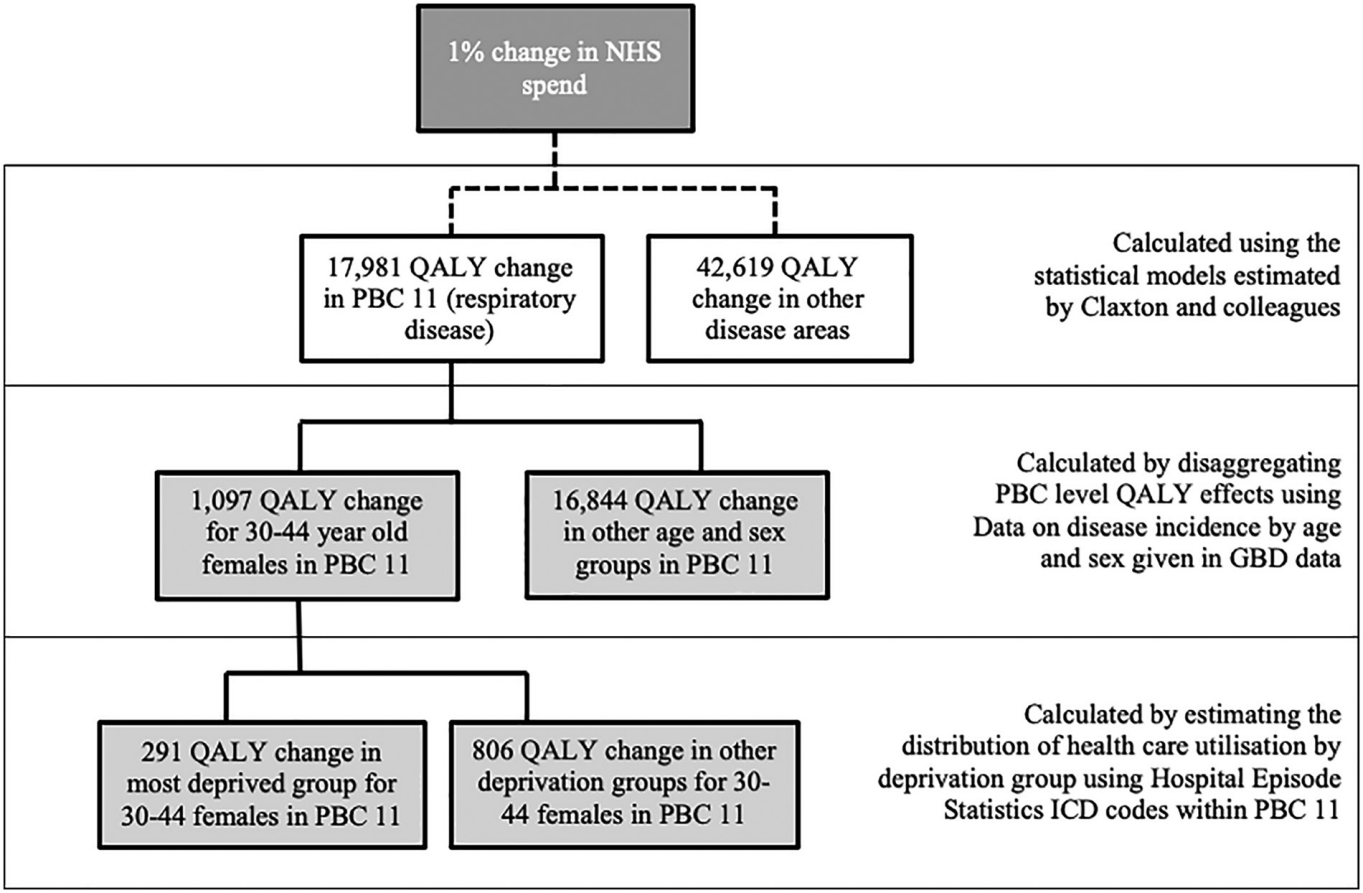
Influence diagram demonstrating how the health effects of a 1% change in health care expenditure for a single disease area are distributed by age, sex, and socioeconomic status. GBD, Global Burden of Disease; ICD, International Classification of Diseases; NHS, National Health Service; PBC, program budgeting category; QALY, quality-adjusted life-year.

These health effects were then allocated to socioeconomic groups using the observed socioeconomic distribution of health care utilization within each age, sex, and disease area group. Summing over disease areas, we obtained the distribution of the overall health effect by age, sex, and socioeconomic status. This process is summarized in Figure 1. Regional effects were also calculated by estimating weighting factors for each of the 326 local authorities (LAs) in England.

### Data and Variables

Hospital Episode Statistics (HES) is a database containing information on all NHS-funded activity in public and private hospitals. Socioeconomic status is assigned to each individual in terms of the deprivation level of place of residence. HES provides a comprehensive and nationally representative data set for our analysis with full coverage of all International Classification of Diseases (ICD) codes.

The primary unit of measurement in HES is the “consultant episode”; patients whose care is transferred between consultants during a single stay in the hospital may have multiple episodes. We used HES data from 2012–2013, the most recent available data at the time of the analysis. Although these data do not temporally align with those used by Claxton and colleagues^9^ (mortality data for 2008–2010 and expenditure 2008–2009), they provided an up-to-date estimate of socioeconomic distributions.

The IMD was used as our measure of socioeconomic status. The IMD is a weighted index of 38 variables covering 7 dimensions of deprivation (employment, income, education, health, crime, living environment, and housing/services) that is given to each of 32482 geographical lower-layer super output areas (LSOAs) in England. Each postcode belongs to an LSOA, giving each patient a deprivation score according to their postcode of residence. The 2004 version of the IMD is provided in HES for the financial year 2013–2014,^24^ which gathers the LSOAs (and their populations) into quintiles (5 equally sized groups) to obtain a 5-level socioeconomic status variable.

Disease is described by ICD codes included as diagnosis variables, of which up to 20 can be recorded for each episode. We converted the 4-digit codes to 3-digit codes, providing 1562 diagnostic categories that were mapped to the 23 PBCs. We related an episode to an ICD code if the latter appeared in any of the 20 diagnosis codes. Consequently, episodes with multiple diagnosis codes were “counted” multiple times.

The HES inpatient data set includes both day cases and overnight stays, encompassing a total of 19578568 unique episodes. We anticipated that the proportion of episodes with missing data would be small and primarily due to administrative or data entry errors. We therefore assumed that data were missing completely at random and removed observations with missing age, sex, or IMD quintile values from the sample as well as those with no diagnosis codes. We further removed observations with sex unspecified.

### Calculating the QALY Distribution

We counted the number of episodes associated with each IMD quintile group within 24992 subgroups (8 age groups, both sexes and 1562 ICD codes). These collapse into 368 subgroups once ICD codes are mapped to the 23 PBCs. This is reduced to 320 subgroups, as 3 PBCs are not allocated any health effects by Claxton and colleagues^9(p103)^ for reasons detailed in their report: Trauma and Injuries (PBC 16), Social Care (PBC 22), and General Medical Services (PBC 23). The count matrices are produced using Stata 12, with all subsequent analyses performed in R.

The counts were converted into proportions to obtain the distribution of utilization by socioeconomic status within each age, sex, and PBC group. Each of the 320 distributions was used to split its respective health effect. One group, 0–5 males in the maternity program (PBC 18), had no episodes associated with it. We assumed a flat socioeconomic distribution for this group (which accounts for less than 0.001 of the total health effect).

To present the results and distributions by equally sized 5-year age groups, we split the 10- and 15-year age groups from the Global Burden of Disease study into 5-year bands, using the respective population proportions from the Office for National Statistics.^25^ For example, 70- to 79-year-old men were disaggregated into the 70 to 74 and 75 to 79 bands according to their general population proportions, which are 0.56 and 0.44, respectively.

### Analyzing Social Inequalities

We analyzed inequalities in the marginal health effects of health care expenditure changes by age, sex, socioeconomic status, PBC, and region. Regional effects were calculated by estimating a weighting factor for each of the 326 LAs in England. The socioeconomic and sex distribution of each LA was used to predict its proportion of health opportunity costs: an LA had a weighting factor greater than 1 when this proportion was greater than its respective share of the overall population and vice versa.

SII and RII were calculated to measure inequalities in the distribution of health effects. A larger negative SII or RII value indicates that a greater proportion of the overall health effect accrues to more deprived groups.

### Sensitivity Analysis

Three types of sensitivity analysis were conducted on the results. First, we reestimated results using the distributions of unique patients within each PBCs instead of episodes. Using episode counts assumes that every episode within each age, sex, and ICD group is associated with an equal probability of generating a QALY regardless of socioeconomic group, whereas using patient counts assumes that each patient has an equal probability of generating a QALY regardless of how many health care episodes they receive. We associated a patient with an ICD code if it appeared in the diagnosis codes of any of their 10 most coded episodes (i.e., those with the highest number of diagnosis codes entered). A second sensitivity analysis was conducted by repeating our analysis using HES episode counts from the preceding 2 years to test whether there were differences in inequality over time.

Although HES provides comprehensive coverage of inpatient secondary care utilization by age, sex, socioeconomic status, and disease, it may not be the most appropriate data source from which to estimate socioeconomic distributions for some disease areas in which secondary care represents a small proportion of health care activity. We were unable to access fully comparable data outside secondary care and thus performed a third sensitivity analysis that compared the socioeconomic gradient for the disease areas targeted in the Quality and Outcomes Framework (QOF) data set to a matched subset of disease areas from HES. The QOF data set^26^ describes the prevalence of selected diseases in the practice population of general practitioners, which allows us to link socioeconomic variables by using the Attribution Dataset on GP Registered Populations to link to LSOA and to IMD. A full description of this sensitivity analysis is reported in Supplementary Appendix D.

## Results

### Descriptive Statistics

Descriptive statistics for HES are reported in Table 2. In total, 119569 (0.006%) observations were excluded from the sample. Another 51344 were deleted as suspected duplicates, leaving a remaining sample size of 19407655 episodes covering a total patient population of 8882110.

**Table 2:** Descriptive Statistics for Hospital Episode Statistics, 2012–2013

Variable	Patients	% Sample	Episodes	% Sample
Total	8882 110	100%	19407655	100
Age, years				
0–4	999334	11.3	1463253	7.5
5–14	363592	4.1	564144	2.9
15–29	1183033	13.3	2141345	11.0
30–44	1427015	16.1	2642378	13.6
45–59	1520374	17.1	3297482	17.0
60–69	1204898	13.6	2983189	15.4
70–79	1143281	12.9	3201919	16.5
80+	1040583	11.7	3113945	16.0
Gender				
Male	3896899	43.9	8826 364	45.5
Female	4985211	56.1	10581291	54.5
IMD				
1 (most deprived)	2090295	23.5	4530 436	23.3
2	1799620	20.3	3998631	20.6
3	1804243	20.3	4018339	20.7
4	1641355	18.5	3571730	18.4
5 (least deprived)	1546597	17.4	3288519	16.9

IMD, Index of Multiple Deprivation.

Females accounted for a larger proportion of patients (56.1%) than males. A near-identical socioeconomic gradient in both episode and patient counts was found. The number of episodes attributed to each PBC and IMD quintile group are provided in Supplementary Table S1. The ratio of episodes in the most deprived to least deprived groups ranged from 0.95 for cancers and tumors (PBC 2) to 2.87 for neonates (PBC 19).

### Main Findings

Table 3 demonstrates how the health benefits of a £50 million budget increase would be distributed between age, sex, and socioeconomic subgroups using Claxton and colleagues’ estimate of marginal NHS productivity of 1 QALY per £12937. Of the 3865 QALYs generated from the increase, nearly twice as many accrued to the most deprived fifth (1019) as compared with the least deprived (537), whereas 25% of the health accrued to those younger than 20 years.

**Table 3:** Distribution of Quality-Adjusted Life-Years by Age and Index of Multiple Deprivation (IMD) Quintile Group for a £50 Million Change in the English National Health Service Budget

	IMD Quintile Group					
Age band, years	1	2	3	4	5	Total
0–4	105	81	79	51	46	362
5–9	44	36	36	25	24	165
10–14	43	35	35	24	24	161
15–19	74	59	58	37	32	260
20–24	82	65	64	41	35	287
25–29	83	66	65	42	35	291
30–34	48	39	38	24	20	169
35–39	46	37	36	23	19	160
40–44	51	41	41	25	21	180
45–49	74	62	61	43	37	277
50–54	66	55	55	39	33	249
55–59	58	48	48	34	29	216
60–64	58	51	51	45	38	243
65–69	54	47	48	42	35	225
70–74	47	43	43	41	36	210
75–79	39	36	36	34	30	174
80–84	24	24	24	25	23	121
85+	24	23	23	24	22	116
Female	541	461	456	337	291	2086
Male	478	387	386	282	246	1779
Total	1019	847	843	620	537	3865

IMD1, most deprived; IMD5, least deprived. An estimate of 1 quality-adjusted life-year (QALY) per £12937 from Claxton et al.^8^ is used to predict the expected number of QALYs (£50 million/£12937 = 3865).^9^

**Figure 2:**
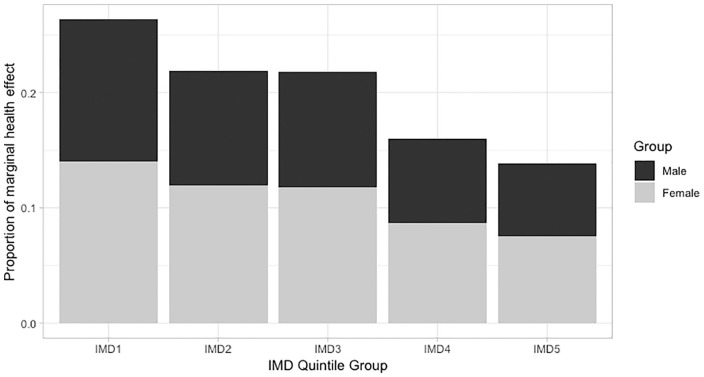
Socioeconomic distribution of health effects from health care expenditure changes for the English population. IMD, Index of Multiple Deprivation (1 = most deprived group, 5 = least deprived). differences in quality-adjusted life-year effects between sexes should be treated with caution. This is because the larger effects for women reflect their levels of health care utilization rather than any systematic differences in the health care services being affected by expenditure changes.

The distribution of health effects by deprivation quintile group is given in Figure 2. The most deprived fifth bore 26.4% of the overall health effect, compared with 13.9% for the most deprived fifth. This disparity is summarized with a SII of −0.15 and an RII of −0.77. For each IMD quintile group, females had a greater share of the health effect. However, the relative differences between deprivation groups were greater for men, with an RII of −0.80 compared with −0.75 for women.

The socioeconomic gradient was most pronounced in younger age bands; a large social gradient is clear from birth until the 40 to 44 band. RII values were consistently around −1.0 up to this age, indicating that the changes in health for the most deprived group is twice the magnitude of those for the least deprived group. Thereafter, disparities reduce to a minimal level: the RII for the 85+ group is −0.06.

#### Inequality within PBCs

Table 4 shows the contribution of the each PBC to overall inequality in health effects. The respiratory program, within which nearly 30% of health effects accrue, exhibits average levels of inequality, with an RII of −0.86. Mental health is one of the most unequal programs, with an RII of −1.28, and cancer is the only prorich program, with an RII of 0.08.

**Table 4:** Inequality in QALY Effects by PBC

PBC	QALY Proportion	QALYs from £50m Spend Increase	SII	RII
Total	1	3865	−0.1542	−0.77
Respiratory	0.297	1146	−0.0509	−0.86
Neurologic	0.141	545	−0.0218	−0.77
Circulation	0.139	539	−0.0152	−0.54
Mental health	0.123	476	−0.0315	−1.28
Endocrine	0.078	303	−0.0129	−0.82
Gastrointestinal	0.057	219	−0.0071	−0.63
Cancers and tumors	0.034	132	0.0005	0.08
Musculoskeletal	0.030	116	−0.0021	−0.35
Blood disorders	0.028	109	−0.0047	−0.83
Infectious diseases	0.020	78	−0.0029	−0.72
Hearing	0.018	70	−0.0014	−0.40
Genito-urinary	0.014	53	−0.0015	−0.56
Dental	0.009	34	−0.0013	−0.74
Vision	0.005	21	−0.0004	−0.36
Skin	0.003	10	−0.0002	−0.45
Poisoning	0.001	4	−0.0002	−0.85
Learning disability	0.001	3	−0.0002	−1.11
Healthy individuals	0.001	3	−0.0003	−1.60
Maternity and neonate	0.001	2	−0.0001	−1.31

PBC, program budgeting category; QALY, quality-adjusted life-year; RII, relative index of inequality; SII, slope index of inequality.

#### Inequality within regions

**Figure 3:**
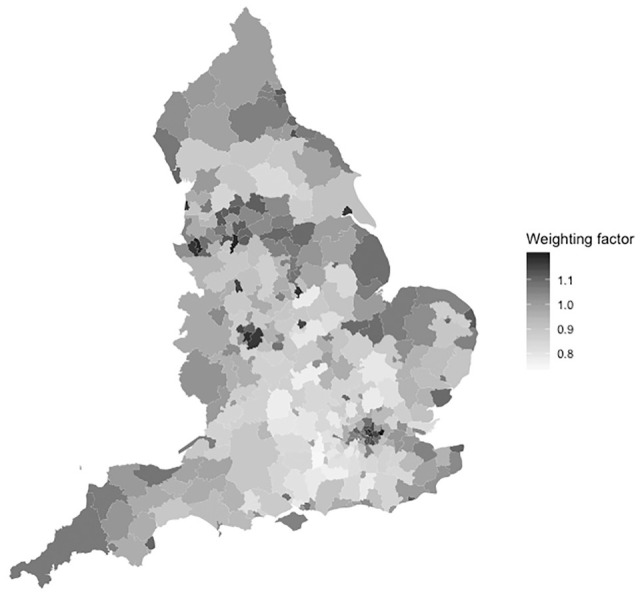
Health effect weighting factors for English local authorities. Weights greater than 1 indicate that health opportunity costs are greater than the expected share based on population alone.

The regional weighting factors for English LAs are shown in Figure 3. LAs in the south and south east generally have a proportion of health effects relative to their population size, with city-based authorities exhibiting larger shares: many London boroughs, as well as Manchester, Birmingham, and Liverpool, have estimated weighting factors greater than 1.15, reflecting a higher share of disadvantaged areas.

### Sensitivity Analysis

Results from all sensitivity analyses are reported in Table 5. The use of unique patient counts reduced socioeconomic inequality in the health effects of health care expenditure, with RII falling from −0.771 to −0.702. Negligible differences were found when using the HES data sets from 2011 or 2012, with the socioeconomic distribution of effects over IMD quintile groups staying consistent over time.

**Table 5:** Summary of Sensitivity Analysis around the Socioeconomic Distribution of Health Effects from Health Care Expenditure: 1) Previous Years’ HES Episode Counts, 2) HES Patient Counts, and 3) QOF Prevalence Data

	IMD Quintile Group					Inequality	
Data Source	1	2	3	4	5	SII	RII
Complete analysis							
Episodes 2012–2013	0.2636	0.2193	0.2180	0.1603	0.1388	−0.1542	−0.7711
Episodes 2011–2012	0.2641	0.2194	0.2178	0.1606	0.1383	−0.1552	−0.7761
Episodes 2010–2011	0.2651	0.2188	0.2184	0.1598	0.1378	−0.1568	−0.7842
Patients 2012–2013	0.2601	0.2161	0.2138	0.1643	0.1457	−0.1403	−0.7015
QOF subset analysis							
Episodes 2012–2013	0.1045	0.0829	0.0811	0.0565	0.0488	−0.0689	−0.9216
QOF 2013–2014	0.1066	0.0848	0.0703	0.0616	0.0505	−0.0677	−0.9051

IMD, Index of Multiple Deprivation; HES, Hospital Episode Statistics; QOF, Quality and Outcomes Framework; SII, slope index of inequality; RII, relative index of inequality. IMD1 indicates most deprived, IMD5, least deprived. Episodes and patient counts estimated using secondary care data; QOF prevalence from primary care. The QOF subset analysis includes only health effects attributable to diseases covered by the QOF data set (approximately 37% of the total health effect). As a result, these rows sum to approximately 0.37 rather than 1.

The inequality in health effects when using prevalence rates from QOF was fractionally smaller than when using utilization statistics from HES. For the 37% of health effects covered by the diseases in the QOF data, RII is −0.905 when using QOF, compared with 0.922 when using HES.

## Discussion

### Main Findings

Our analysis shows how the health effects of changes in government health expenditure can be disaggregated by equity-relevant social groups. Applying our framework to the results of a study of the English NHS, we found that expenditure changes imposed greater health impacts on the most socioeconomically deprived and were concentrated in younger age groups. The results support the conclusions of both Asaria et al.^27^ and Barr et al.^28^ that increases in NHS funding during the 2000s^29^ likely contributed to a reduction in socioeconomic health inequalities.

Our case study results are underpinned by evidence produced by Claxton and colleagues on the relationship between local health expenditure and mortality, which they combined with other data to estimate the marginal effects of NHS expenditure on population QALYs. A full list of these assumptions underpinning their results is given in table 32 of their report,^9(p83)^ upon which critiques and responses have subsequently been published.^30–32^ When the plausibility of these assumptions was tested against expert clinical judgment, the results suggested that they are likely to be on the conservative side and will lead to an underestimate of marginal productivity (or an overestimate of the cost per QALY at the margin).^33^ Subsequent analysis of English data using different statistical models has also yielded consistent results over time^20,21^ and support the estimates used in our case study as being genuine causal effects. Sensitivity analyses suggest that our analysis is robust to quirks in health care utilization specific to 2013, to the use of secondary care utilization data rather than prevalence data or primary care utilization data, and that our findings reflect consistent socioeconomic patterns by disease.

In the same way that the results of an analysis of marginal productivity can be used to estimate the health opportunity cost of health system investment decisions, the results derived from our framework can similarly be used to provide a distribution of health opportunity costs between equity-relevant groups. This is of particular use in distributional cost-effectiveness analyses that look at the differences in costs and effects by social group.^10,34,35^ The metric of interest in this type of analysis is the distribution of net health benefit: the health benefits of a new technology minus the health opportunity costs of forgoing investment in other interventions. The adoption of a single intervention can usually be considered marginal (relative to the full set of resources) and will therefore not affect the marginal productivity of the health system. This means that the estimates generated by our approach can be employed in all subsequent economic evaluations that impose costs on the health sector budget. This is consistent with the way in which health opportunity costs are currently incorporated into the approaches of agencies such as National Institute for Health and Care Excellence in England.^36^

### Limitations and Assumptions

The framework proposed in this article is needed because of the absence of health system marginal productivity studies that directly estimate health effects by social groups. Indirectly estimating this distribution requires us to combine the results of available marginal productivity studies with data on the social distribution of health care use.

The validity of the results is reliant on the quality of the original marginal productivity study. Analysis of marginal productivity presents a range of challenges with regard to estimating causal effects, namely, availability of good-quality data, endogeneity bias in the statistical model, and time lags between expenditure and health outcome. Although broader application of this framework is limited by the availability of good-quality studies that estimate the marginal productivity of health care expenditure, suitable estimates are increasingly available.^13–15,20^

Using health care utilization data to disaggregate health effects assumes that the health impact of 1 episode of healthcare is uniform across social groups. Although empirical work suggests that the health outcomes from health care are generally better for less deprived groups,^37^ little work has been conducted on the direct link between health care inputs and health outputs by socioeconomic group. Those in more deprived areas may be more likely to seek care only when more severely ill and could therefore have a higher capacity to benefit from treatment, although treatment effectiveness may itself be reduced if patients present later. Furthermore, more affluent groups may be more effective at producing health from any given input of public sector resource, because of lesser comorbidity and greater ability to invest additional time and resources in recovery, care coordination, and prevention. To the extent that the latter effect dominates, our results may overestimate the gradient in health effects.

Methods for fully characterizing the uncertainty are not covered in this article. Uncertainty over the proportions that characterize the social distributions of health care utilization must also be combined with uncertainty around the health effect proportions from the marginal productivity analysis. In principle, this could be propagated through all analytic steps via Monte Carlo simulation but were not incorporated in our case study.

How representative the secondary factor is for capturing the impact of expenditure on outcomes requires consideration. We use secondary care utilization data, and although many diseases are treated in secondary care, some disease areas or conditions are principally treated in primary care, such as asthma, or in specialist facilities, such as schizophrenia or other mental health conditions. We could not obtain primary care utilization data that could test this hypothesis in our case study, although using primary care data from QOF indicates that results were largely comparable to secondary care utilization. However, these prevalence data do not account for patterns of utilization and would not capture the additional health benefits that sicker patients in more deprived groups obtain from multiple visits to primary care, for example.

### Implications and Further Research

An important application of our results is their use in health technology assessment. The numbers in Table 3 are interpretable as the distribution of health opportunity costs that result from not funding £50 million worth of alternative health services. This distribution can help to inform decision makers on what impact future interventions have on health inequality. This could be through informal consideration or a distributional cost-effectiveness model,^10,34^ in which our estimates can be combined with an equivalent distribution of health benefits generated by an intervention.^38^

There is scope to improve our estimates by using better data with which to estimate the socioeconomic distributions of health care utilization. For example, other data sets such as the Clinical Practice Research Datalink and the Mental Health Minimum Dataset could provide socioeconomic distributions of relevant conditions by age and sex in primary care and specialist mental health centers, respectively. Future research should also investigate the differences in health benefit achieved from receiving health care, which our analysis has assumed is the same for all socioeconomic groups. Lastly, similar analyses should be conducted for social care expenditure, because a comparison between the marginal effects of expenditures of health and social care can help inform resource allocation priorities with respect to health inequalities.

The framework demonstrated in this study provides additional evidence to decision makers on the distributional effects of health expenditure compared with traditional benefit incidence studies and can contribute toward the reduction of unfair population health inequalities.

## Supplemental Material

appendices_online_supp – Supplemental material for Estimating Social Variation in the Health Effects of Changes in Health Care Expenditure Click here for additional data file. Supplemental material, appendices_online_supp for Estimating Social Variation in the Health Effects of Changes in Health Care Expenditure by James Love-Koh, Richard Cookson, Karl Claxton and Susan Griffin in Medical Decision Making
