# Supplementary material for: Estimating Social Variation in the Health Effects of Changes in Health Care Expenditure
Source: Med Decis Making. 2020 Feb 15;40(2):170–82. doi: 10.1177/0272989X20904360 (PMC7430104; doi:10.1177/0272989X20904360)
Supplement: appendices_online_supp – Supplemental material for Estimating Social Variation in the Health Effects of Changes in Health Care Expenditure [file appendices_online_supp.docx]

# Appendix A – Worked example of basic framework for disaggregating health effects by social group

This stylized example provides the basic intuition behind the framework presented in the main paper. Recall that the central equation for the health effect of a change in health expenditure accruing to social group $\boldsymbol{x}$ is:

$$h_{\boldsymbol{x}}=\sum_{J} h_{\boldsymbol{x}j}=\sum_{J} h_{j}p_{\boldsymbol{x}j}$$

Where $h$ is the health effect in terms of healthy life years, $j$ is a secondary factor linking the health effects to social characteristics and $p$ is a quantity that allocates the health effects to social groups (for example, the proportion of healthcare utilisation).

Consider a health system for which a study has been conducted on the marginal productivity of the health system. The authors estimated that for every $5,000 invested (or disinvested), one healthy life year is gained (or lost). For an investment of $5 million, therefore, 1,000 healthy life years are expected to be gained.

We wish to investigate how these healthy life years are distributed between two social groups – high income and low income. Firstly, a secondary factor is required to link the overall health gains with these social groups. This is available from the marginal productivity study, which estimated the overall health gain by adding together the health effects across three disease areas. Disease 1 contributes 40% of the health gains, Disease 2 contributes 35% and Disease 3 contributes 25%. The quantities $h_{j}$ are therefore 400, 350 and 250 healthy life years, respectively.

The distribution of effects between low and high income groups can be estimated by looking at healthcare utilisation in each of these groups ($p_{\boldsymbol{x}j}$). The calculations are presented in the table below.

|  | **Low income** | **High income** |
| --- | --- | --- |
| *Proportion of healthcare utilisation* | | |
| A. Disease 1 | 0.7 | 0.3 |
| B. Disease 2 | 0.6 | 0.4 |
| C. Disease 3 | 0.4 | 0.6 |
|  |  |  |
| *Healthy life years* | | |
| D. Disease 1 (A x 400) | 280 | 120 |
| E. Disease 2 (B x 350) | 210 | 140 |
| F. Disease 3 (C x 250) | 100 | 150 |
| G. Total (D + E + F) | 590 | 410 |

Rows A to C are the distributions of utilisation for each disease by income ($p_{\boldsymbol{x}j}$). Rows D to F are the distributions of health effects by disease and income ($h_{\boldsymbol{x}j}$). Row G is the distribution of health effects by income only ($h_{\boldsymbol{x}}$). This provides a split of marginal health of 59% to the low income group and 41% to the high income group. These proportions can be used to allocate any other marginal health effect. For example, if the investment was instead $1 million (implying a change of 200 healthy life years), the health gains for the low and high income groups would be 118 and 82 healthy life years, respectively.

# Appendix B – Worked example for imputing regional opportunity costs

Estimates of the socioeconomic and demographic distribution of the health benefits of marginal changes in healthcare expenditure can be used to impute geographical distributions. This is done by linking geographical areas with the socioeconomic and demographic characteristics. For example, if low socioeconomic status individuals incur a higher share of health effects, then more socioeconomically deprived geographical areas should also incur a higher share. We demonstrate the calculations in a worked example for Manchester, one of the 326 local authorities (LAs) in England included in our analysis.

First the distribution of marginal health effect (by IMD and sex) is divided through by the respective population estimate to obtain a notional ‘per person’ distribution of health effect. These are then scaled to the population of the Manchester LA. This provides the proportion of the overall health effect that can be expected to fall on those living within the LA based purely on its socioeconomic and demographic characteristics. These are shown in the table below.

|  | **IMD1** | **IMD2** | **IMD3** | **IMD4** | **IMD5** |
| --- | --- | --- | --- | --- | --- |
| *A. Marginal health effect distribution* | | | | | |
| Male | 0.124 | 0.100 | 0.100 | 0.073 | 0.064 |
| Female | 0.140 | 0.119 | 0.118 | 0.087 | 0.075 |
|  |  |  |  |  |  |
| *B. Population - England* | | | | | |
| Male | 5,567,632 | 5,660,089 | 5,482,431 | 5,364,885 | 5,225,883 |
| Female | 5,671,611 | 5,721,941 | 5,607,885 | 5,531,034 | 5,434,676 |
|  |  |  |  |  |  |
| *C. Population - Manchester* | | | | | |
| Male | 159,766 | 81,155 | 22,792 | 10,217 | 722 |
| Female | 159,581 | 74,999 | 21,439 | 9,810 | 782 |
|  |  |  |  |  |  |
| *D. Marginal health effects - Manchester (C*(A/B)* | | | | | |
| Male | 0.00355 | 0.00143 | 0.00042 | 0.00014 | 0.00001 |
| Female | 0.00394 | 0.00156 | 0.00045 | 0.00015 | 0.00001 |

The weighting factor can then compare the proportion of the overall health effect accruing to those in the LA with its share of the general population. In the example above, Manchester’s share of the health effects (0.0117) is greater than its share of the population (0.0098), yielding a weighting factor of 1.191.

# Appendix C – Description of Claxton et al. study estimating the effect of NHS expenditure on quality-adjusted life years

## Effect of expenditure on mortality

Since 2003, each regional spending body of the English NHS (formerly Primary Care Trusts, now Clinical Commissioning Groups) has been required to categorise all expenditure into one of 23 programme budgeting categories (PBC). Each PBC covers a broad clinical area such as cancer or infectious disease, and is defined by a subset of International Classification of Disease (ICD) Version 10 codes. Martin et al. showed how observations on expenditure could be linked to mortality using routine Primary Care Trust level data within each PBC [5],[16] Claxton and colleagues built on this analysis using more recent data that included all 152 Primary Care Trusts and covered all programmes of care.

Analysis of cross-sectional data can potentially suffer from endogeneity bias; for example, reverse causality if poor health outcomes motivate decision-makers to increase health expenditure. These and other problems may account for the substantial variation in published estimates of the magnitude of the health effect of additional health care expenditure [2]-[7]. Claxton and colleagues therefore use a two-stage least squares instrumental variables approach to account for endogeneity. Two equations are estimated for each PBC: an expenditure equation linking the NHS budget to PBC expenditure and an outcome equation linking PBC mortality to PBC expenditure in a particular year. The equations control for need by including the Department of Health’s formula for regional need and/or programme-specific variables (such as diabetes prevalence rates). Three-year averages of mortality are used to account for temporal fluctuations, the first year of which aligns with that of the expenditure data, thereby allowing for a lagged effect of the latter on the former to be captured. Since mortality data are only available for eleven PBCs, the productivity of the remainder are assumed to be equal to the average of those where health effects could be estimated (with the exception of PBC 23, 'other', which is assumed to have zero health gain). The expenditure and outcome equations, respectively, are as follows:

$x_{i}=\alpha+\beta n_{i}+\gamma m_{i}+\theta y_{i}+\varepsilon_{i}$ for $i=1,\ldots,15$2

$h_{i}=\rho+\delta n_{i}+\pi x_{i}+\varepsilon_{i}$ for $i=1,\ldots,15$2

where $x_{i}$ is expenditure; $n_{i}$ is the own programme need for care; $m_{i}$ is the need for care in other programmes; $y_{i}$ is the total budget and $h_{i}$ is the health gain in PCT $i$. The variables are log-transformed such that the coefficients of interest, $\theta$ and $\pi$, represent elasticities: $\theta$ is the percentage change of a PBC budget with respect to a percentage change in the overall NHS budget; $\pi$ is the percentage change in health for a percentage change in a PBC budget.

To account for the endogeneity in both equations, a large number of instruments are acquired from census data and tailored to each PBC. A battery of tests, including the Hansen–Sargen test, the Kleibergen–Paap Lagrange multiplier test and the Kleibergen–Paap F-statistic, are performed; where instruments are weak or invalid, a combination of other census-derived variables are used instead. A full list of all the instruments considered by the authors is given in Table 92 of their report [11, p. 347]. This strategy ensured that, even though endogeneity is indeed found to be present in many of the expenditure and outcome equations, the instrument set used for each is valid and sufficiently strong, thereby giving consistently estimated coefficients. The instruments included for each expenditure and outcome equation and their performance in the tests are detailed in Appendix 2 of Claxton and colleagues’ report [11, p. 314].

## Extending outcome to QALYs

To estimate the effect of spending on QALYs, mortality effects are first converted into ‘net’ years of life lost by disease area using data on age of death by PBC from the Office for National Statistics (ONS). This accounts for counterfactual deaths that would have occurred in the at-risk populations; Chapter 4 of their report provides full details of the calculations [11, p. 45]. When the elasticities from the mortality equations are applied to net years of life lost, they provide an estimate of life years gained from additional expenditure.

Claxton and colleagues then translate these into QALYs by adding the health lost due to reduced quality of life whilst living with a disease to the health lost due to premature death, using data at the ICD code level. The process is described in Chapter 4 of their report [11, p. 56] and involves weighting years of life lived with, and lost to, each disease by combining evidence on net years of life lost, incidence, duration of disease, age and gender with quality of life scores by disease. The QALY burdens for each ICD code within a PBC are then summed to generate a PBC level QALY burden. The change in QALYs for a change in expenditure is then yielded by applying the proportionate effect of spend on mortality (i.e. the spend elasticity multiplied by the outcome elasticity) to the QALY burden for a given PBC. This implies that the ratio of PBC-level health effects of life extension to quality of life gains is identical to that in the respective QALY burden.

## Disaggregating health effects by age, sex and disease

The main work by Claxton and colleagues only requires health effects at the PBC level, but a subsequent publication [17] provides more detailed ICD level breakdowns [18]. These data can be re-aggregated back to PBC level to get the proportion of the overall health effect attributable to that PBC (in line with the original analysis). By then assuming that QALY gains are distributed within a PBC population according to the age and gender distribution of the incident population, the authors use disease-specific incidence statistics from the World Health Organization’s Global Burden of Disease Study [19] to disaggregate the PBC-level health effects to age and gender groups.

**Appendix D – Calculating disease prevalence by socioeconomic quintile group using primary care data**

Whilst Hospital Episode Statistics (HES) provides comprehensive coverage of inpatient secondary care utilisation by age, gender, socioeconomic status and disease (measured by International Classification of Disease (ICD) code), it may not be the most appropriate data source from which to estimate socioeconomic distributions for some clinical areas. The socioeconomic patterns observed in inpatient secondary care might not be reliable proxies for how the health benefits accruing to each disease area are distributed, especially for disease areas where the proportion of total healthcare activity taking place in inpatient secondary care is small.

We therefore sought data that provided information on diseases and conditions typically treated in primary care by socioeconomic status (preferably Index of Multiple Deprivation (IMD)). Once such source was the Quality and Outcomes Framework (QOF) dataset, which includes information on socioeconomic (but not age or gender) distribution of diseases at the level of general practitioner (GP) clinic. QOF is an incentive scheme for NHS GPs in the UK that provides financial rewards to each practice for achieving specific clinical goals within their patient population, known as ‘indicators’. Many of the indicators involve ensuring that a sufficient proportion of the practice population with a certain condition receive a test or treatment (for example, the proportion of patients with coronary heart disease who have received an influenza immunisation). This necessitates having an estimate of the at-risk population for each condition of interest for each practice so their achievement can be measured, from which a practice-level prevalence rate can be calculated. These are provided for all conditions relating to the indicators and are published annually [27].

The Quality and Outcomes Framework (QOF) primary care data provides prevalence rates by disease for each GP practice in England. However, the practices do no align with local super output areas (LSOAs) that are used to calculate local area deprivation scores using the Index of Multiple Deprivation, as some practices straddle multiple LSOAs. Therefore we use the Attribution Dataset on GP Registered Populations, which disaggregates each practice population by LSOA. We used the most recent datasets available to us: the Attribution data for 2012 and QOF prevalence statistics for 2013/14.

With these data, we can calculate prevalence by IMD using the following process for each condition:

1. Apply the practice-level prevalence rate to each postcode portion of the practice population, giving the expected number of cases of a condition by practice, disaggregated by postcode;
2. Add the number of expected cases for each postcode (which is split between multiple practices). Each postcode is assigned its IMD score and IMD quintile group;
3. Add up the number of cases for each IMD decile and dividing by the total number of cases to obtain the relative proportions for IMD deciles (i.e. the socioeconomic distribution);

The prevalence rates by QOF condition are not direct substitutes for the HES dataset as they are not defined by ICD code or IMD quintile. We therefore mapped the conditions to their ICD codes (or subset of codes), shown in Table A2.

The QOF distributions are then used to replace the episode distributions extracted from HES, and are applied to all the age-gender-ICD groups that constitute each condition. To investigate the impact of using this alternative source of data, we compared the socioeconomic distribution of the QALY proportions for only the ICD codes covered by the QOF dataset. These 54 codes represent 37.4% of the total health effect. The distribution over IMD quintiles is shown in Table 4 in the main report. The distribution when using QOF estimates was marginally more equal than the HES distributions; RII and SII decreased from -0.922 and -0.0689 for HES to -0.905 and -0.0677 for QOF, respectively.

Table A1: Number of episodes from Hospital Episode Statistics by Index of Multiple Deprivation (IMD) quintile group and Programme Budgeting Category (PBC)

|  |  |  |  | **IMD Quintile Group** | |  |  |
| --- | --- | --- | --- | --- | --- | --- | --- |
|  | **PBC** | **1** | **2** | **3** | **4** | **5** | **Ratio 1/5** |
| 1 | Infectious disease | 73,206 | 65,773 | 65,792 | 55,640 | 51,106 | 1.43 |
| 2 | Cancers and tumours | 798,965 | 769,636 | 790,868 | 875,026 | 838,692 | 0.95 |
| 3 | Blood disorders | 288,574 | 260,946 | 266,963 | 218,611 | 193,114 | 1.49 |
| 4 | Endocrine, nutritional | 977,489 | 888,327 | 880,756 | 738,236 | 623,516 | 1.57 |
| 5 | Mental health | 1,182,758 | 935,190 | 917,735 | 603,987 | 466,905 | 2.53 |
| 6 | Learning disability | 55,110 | 44,280 | 43,390 | 28,797 | 21,897 | 2.52 |
| 7 | Neurological | 389,763 | 343,101 | 339,122 | 293,529 | 262,108 | 1.49 |
| 8 | Vision problems | 259,087 | 246,621 | 248,643 | 255,468 | 233,922 | 1.11 |
| 9 | Hearing problems | 75,419 | 69,193 | 69,268 | 63,735 | 57,986 | 1.30 |
| 10 | Circulatory disease | 2,625,616 | 2,415,341 | 2,422,808 | 2,299,099 | 2,023,139 | 1.30 |
| 11 | Respiratory disease | 1,215,577 | 1,050,923 | 1,046,605 | 840,736 | 710,081 | 1.71 |
| 12 | Dental problems | 79,505 | 74,205 | 72,900 | 50,626 | 43,713 | 1.82 |
| 13 | Gastrointestinal system | 1,288,223 | 1,160,694 | 1,170,376 | 1,051,384 | 954,478 | 1.35 |
| 14 | Skin problems | 287,882 | 255,144 | 251,871 | 213,796 | 186,235 | 1.55 |
| 15 | Musculoskeletal system | 894,024 | 827,430 | 829,136 | 784,789 | 712,146 | 1.26 |
| 17 | Genitourinary system | 1,086,429 | 1,001,856 | 1,001,596 | 860,605 | 740,267 | 1.47 |
| 18 | Maternity | 534,888 | 474,522 | 461,860 | 309,722 | 279,065 | 1.92 |
| 19 | Neonate | 227,315 | 129,349 | 124,651 | 87,885 | 79,231 | 2.87 |
| 20 | Poisoning | 21,984 | 18,753 | 19,307 | 15,167 | 13,946 | 1.58 |
| 21 | Healthy individuals | 340,182 | 313,719 | 305,312 | 242,280 | 209,867 | 1.62 |
|  | **Total** | **12,701,996** | **11,345,003** | **11,328,959** | **9,889,118** | **8,701,414** | **1.46** |

**Note**s:

IMD 1 = most deprived; IMD 5 = least deprived

Episodes are attributed to a PBC/IMD group if they appear in any of its respective diagnosis codes. Counts therefore exceed the episode total of 19,407,655

Table A2: ICD codes covered by the disease included in the Quality and Outcomes Framework dataset

| **Disease** | **ICD Codes** |
| --- | --- |
| Asthma | J45 |
| Atrial Fibrillation | I48 |
| CHD | I20-25 |
| CKD | N18 |
| COPD | J40-44, J47 |
| Dementia | F00-F07 |
| Depression | F32 |
| Diabetes | E10-14 |
| Epilepsy | G40-41 |
| Heart Failure | I50 |
| Hypertension | I10-15 |
| Learning disability | F82 |
| Obesity | E66 |
| Osteoperosis | M81-82 |
| PAD | I73 |
| Rheumatoid Arthritis | M05-06 |
| Stroke | I61-64, G45 |
| Hypothyroidism | E00-07 |
